# Supplementary material for: Polymorphisms in Pfkelch13 domains before and after the introduction of artemisinin-based combination therapy in Southwest Nigeria
Source: PLoS One. 2025 Mar 31;20(3):e0316479. doi: 10.1371/journal.pone.0316479 (PMC11957316; doi:10.1371/journal.pone.0316479)

Samples: 12316
Bases: 955
Average spacing: 13

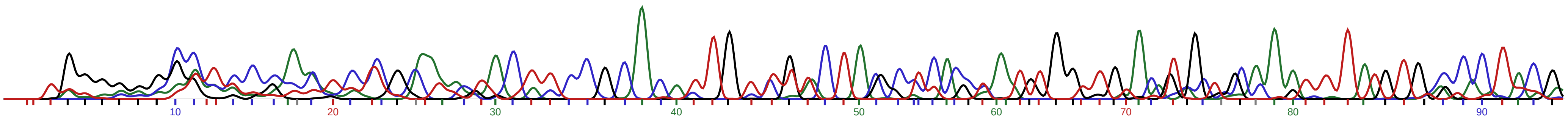

TT T G G G G G G C C TT C C C A - C T C TC G - T A C T A C T T C C G C A C A T T G T T G T C T A C C CC C AC G T AA TGT G GT T G T A C T G C - G - A A T T T A G T G C C C T A C G

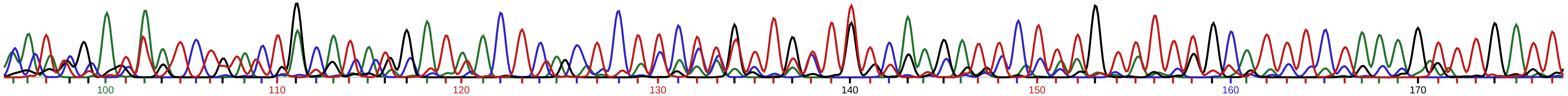

A A T G G A T A G T C T G A C T G C A T A G G A T T A C T C A C T C T T C T T G T T G T T G T C G A G A T T C T T T G T T T T T G C A T T T C T A A A G T T T G A T T

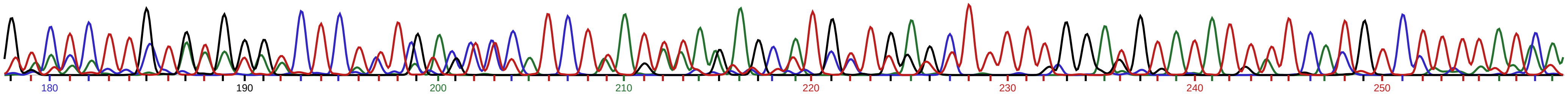

G T C T C T T G T G T G G G T C T C T T T G A G T T C A T C T T A T T T A G A G C A T G T T G A G C T T T T T G G A T G T A T A T T T T C A T G T C T T T T A T C A

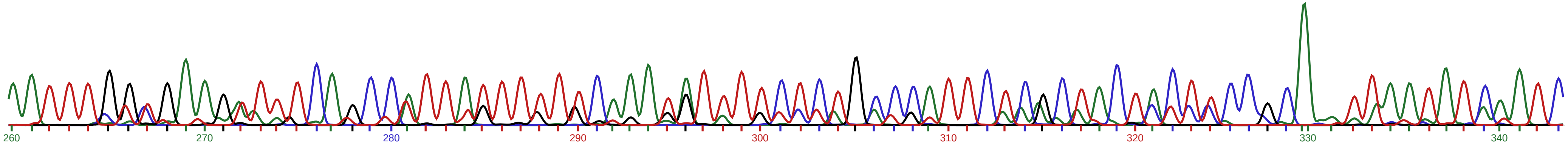

A A T T T G G T G A A G T T T T C A G C C A T T A T T T T T T C A A A T G T T T T C T C T G C C C A T T C T C G C T A C T A C T T C C G C A A A T T A A T A T C A A T C

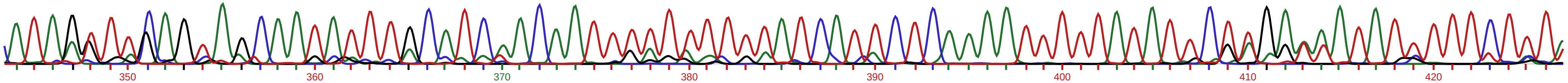

A T A G T T T C A G T A G C A A T A T T T G C A T C A A C A A T T T T T T T T T T A T C A T T C T C A A A A T T T T T A T A T T C T T G A T A A T A T T T T T C T T T

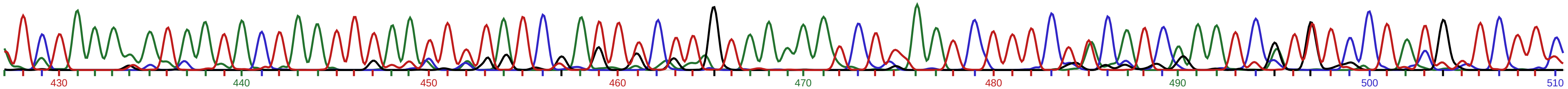

A T C T A A A A A T A A T A C T A A T T T A A T T T T T A T C G A T T T C T T G T A A A A A T C T T A A T C T T T C T T C A T C A A A T C G T G T C C T A T G T T C T T C

Samples: 12316
Bases: 955
Average spacing: 13

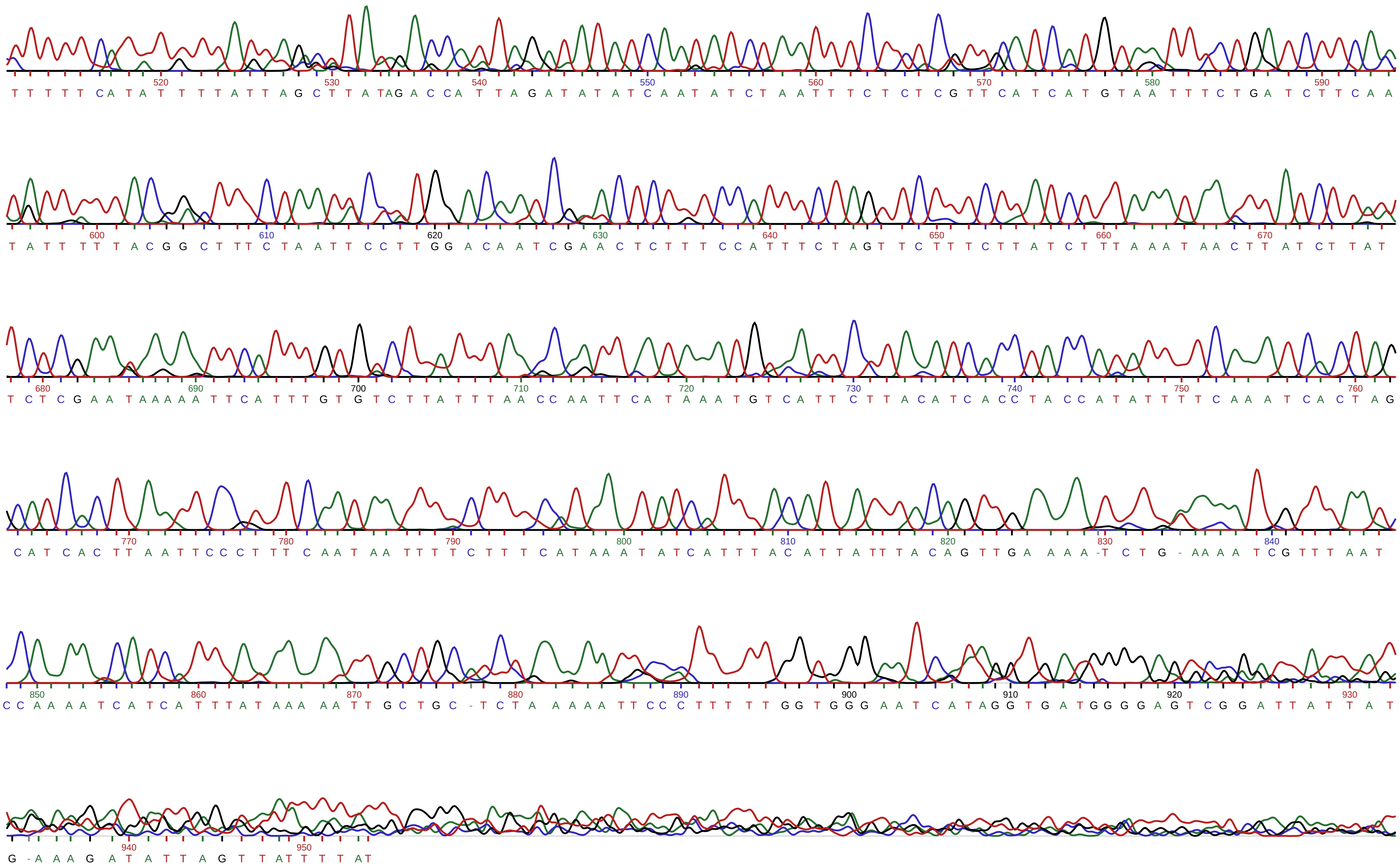

Samples:	12316
Bases:	955
Average spacing:	13

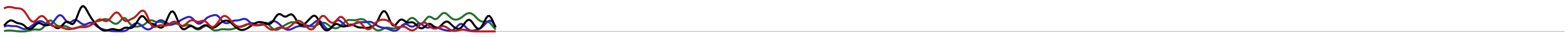

Supplement: Supporting information 2 — (ZIP) [file pone.0316479.s002.zip › 201KN2R_PREMIX_Plate_KELCH2_H10.pdf]
